# Synthesis, Characterization, and *In Vitro* Studies of an Reactive Oxygen Species (ROS)-Responsive Methoxy Polyethylene Glycol-Thioketal-Melphalan Prodrug for Glioblastoma Treatment

**DOI:** 10.3389/fphar.2020.00574

**Published:** 2020-05-04

**Authors:** Natalia Oddone, Frank Boury, Emmanuel Garcion, Andreas M. Grabrucker, M. Carmen Martinez, Federica Da Ros, Anna Janaszewska, Flavio Forni, Maria Angela Vandelli, Giovanni Tosi, Barbara Ruozi, Jason T. Duskey

**Affiliations:** ^1^Nanotech Lab TeFarTI Group, Department of Life Sciences, University of Modena and Reggio Emilia, Modena, Italy; ^2^CRCINA, INSERM, Université de Nantes, Université d’Angers, Angers, France; ^3^Department of Biological Sciences, University of Limerick, Limerick, Ireland; ^4^Bernal Institute, University of Limerick, Limerick, Ireland; ^5^Health Research Institute (HRI), University of Limerick, Limerick, Ireland; ^6^SOPAM, U1063, INSERM, UNIV Angers, SFR ICAT, Angers, France; ^7^Department of General Biophysics, Faculty of Biology and Environmental Protection, Lodz, Poland; ^8^Umberto Veronesi Foundation, Milano, Italy

**Keywords:** glioblastoma, ROS-responsive prodrug, melphalan, TK-technology, X-ray, radiotherapy

## Abstract

Glioblastoma (GBM) is the most frequent and aggressive primary tumor of the brain and averages a life expectancy in diagnosed patients of only 15 months. Hence, more effective therapies against this malignancy are urgently needed. Several diseases, including cancer, are featured by high levels of reactive oxygen species (ROS), which are possible GBM hallmarks to target or benefit from. Therefore, the covalent linkage of drugs to ROS-responsive molecules can be exploited aiming for a selective drug release within relevant pathological environments. In this work, we designed a new ROS-responsive prodrug by using Melphalan (MPH) covalently coupled with methoxy polyethylene glycol (mPEG) through a ROS-cleavable group thioketal (TK), demonstrating the capacity to self-assembly into nanosized micelles. Full chemical-physical characterization was conducted on the polymeric-prodrug and proper controls, along with *in vitro* cytotoxicity assayed on different GBM cell lines and “healthy” astrocyte cells confirming the absence of any cytotoxicity of the prodrug on healthy cells (i.e. astrocytes). These results were compared with the non-ROS responsive counterpart, underlining the anti-tumoral activity of ROS-responsive compared to the non-ROS-responsive prodrug on GBM cells expressing high levels of ROS. On the other hand, the combination treatment with this ROS-responsive prodrug and X-ray irradiation on human GBM cells resulted in an increase of the antitumoral effect, and this might be connected to radiotherapy. Hence, these results represent a starting point for a rationale design of innovative and tailored ROS-responsive prodrugs to be used in GBM therapy and in combination with radiotherapy.

**Graphical Abstract:**
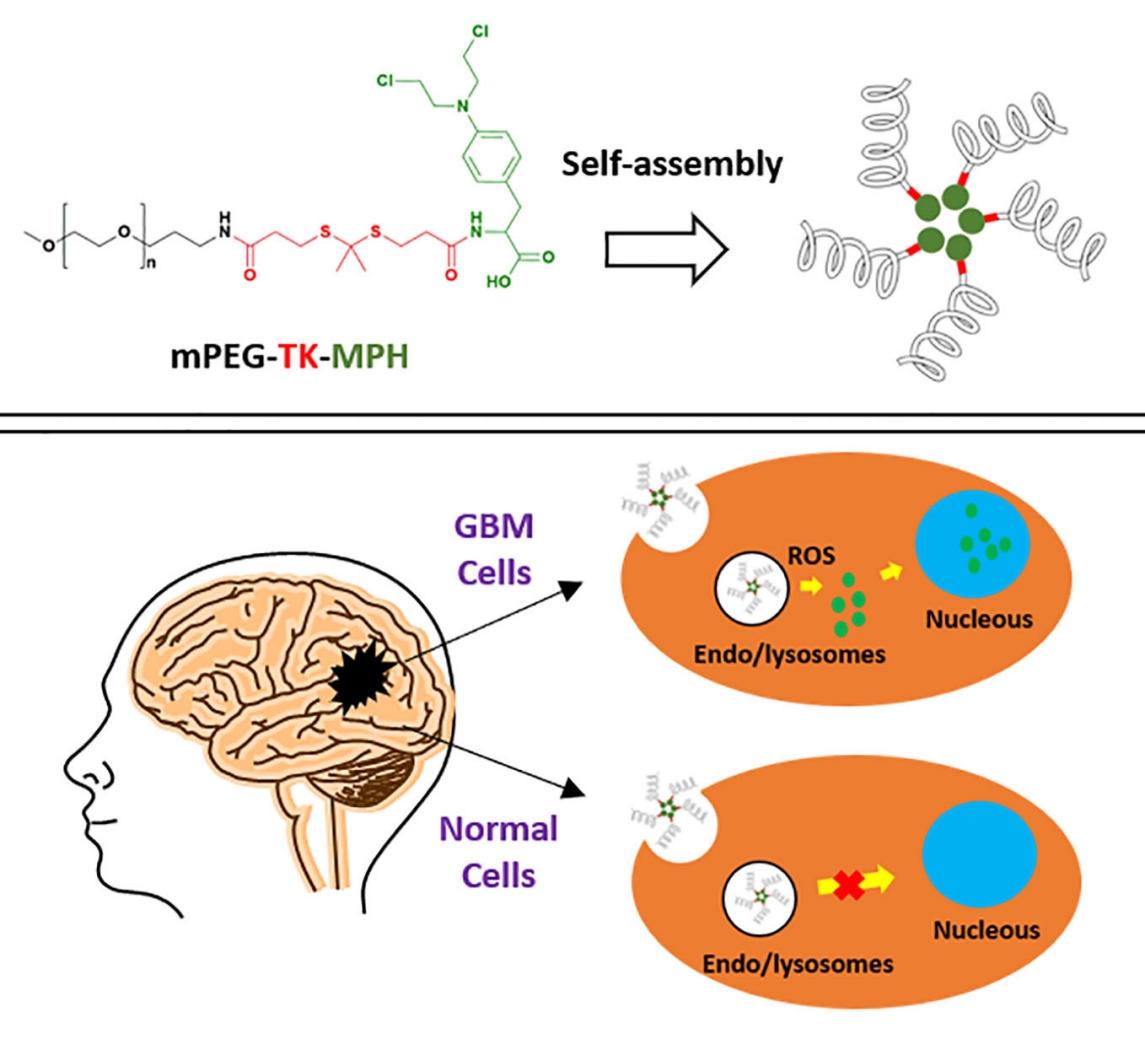


## Introduction

Cancer represents one of the most lethal diseases worldwide (Kim et al., 2014), and in high-income countries, the number of cancer deaths is currently double the deaths caused by cardiovascular disease (CVD) (Mahase, 2019). Among all deadly types of cancer, brain cancer is one of the most difficult to treat and cure (Lee et al., 2013). In particular, glioblastoma (GBM) malignant grade IV astrocytic tumor (Resende et al., 2018) is the most frequent and aggressive primary tumor with the poorest prognosis (Tsai et al., 2012; Gramatzki et al., 2013; Lee et al., 2013; Legendre et al., 2016; Yang et al., 2016) with a median survival time of only 15 months (Majewska et al., 2017). Due to the ability of GBM cells to migrate to other brain regions, after diagnosis and initial treatment, GBM recurrence takes place within 6–12 months after diagnosis (Tsai et al., 2012; Lee et al., 2013; Yang et al., 2016) as a secondary tumor, which is one of the main reasons that account for the lethality of GBM (Haar et al., 2012).

Standard GBM therapy consists of tumor surgery followed by concomitant radio- and chemotherapy with temozolomide (TMZ) (Majewska et al., 2017; Resende et al., 2018), one of the few chemotherapeutic agents with an acceptable blood-brain barrier (BBB) penetration (20% of the injected dose). Apart from TMZ, carmustine, and lomustine are the most widely used drugs for GBM treatment (Tseng et al., 2015). Nevertheless, lomustine shows a brain/plasma ratio of 20% in rats, which is lower compared to TMZ, with 22–41%. Regarding carmustine, its passage through the BBB is lower with higher CNS toxicity (Wang et al., 2019a). Bevacizumab is used in patients that do not respond to TMZ, but its poor BBB crossing leads to high concentrations being administered and, consequently, to adverse effects (Sousa et al., 2019).

Nevertheless, standard therapy with TMZ only increases survival for 2.5 months (Resende et al., 2018). This is probably due to the aggressiveness of recurrent tumors, the antitumoral drug resistance frequently observed (Raucher et al., 2018), and the often low selectivity of chemotherapeutics (Hagen et al., 2012), with frequent administrations (Farokhzad and Langer, 2006) leading to decreased patient compliance and increase in drug resistance (Yang et al., 2016). Hence, the design of new therapies against GBM that prolong survival or cure the disease are strongly needed.

Drug Delivery Systems (DDS) that improve residence time in circulation, solubility, and targetability of chemotherapeutic drugs were applied for some products on the market such as Abraxane^®^, Doxil^®^, Onivyde^®^, and Zoladex^®^ that are already approved for their clinical use in cancer treatment (Farokhzad and Langer, 2006; Liu et al., 2016; Gonda et al., 2019).

Nowadays, cancer therapy strategies are aiming to be more personalized due to the presence of tumor heterogeneity among cancer patients (Meel et al., 2019) leading to a possible inefficacy of designed approaches. Thus, in order to create more suitable DDS for the treatment of cancer, it is necessary to develop more precise nanomedicines that take into consideration tumor biology and peculiar pathological features (Gonda et al., 2019). In this regard, a DDS with the possibility to sense the tumor environment for a more selective drug release (Cobo et al., 2015) holds great promise when it comes to advances in the development of so-called “Smart” DDS.

Aiming at drug release in a given pathological condition is highly sought after (Najer et al., 2015; Duskey et al., 2017; Rigon et al., 2019; Tosi et al., 2019), and “Smart” DDS that are sensitive to a specific stimulus (such as pH, enzymes, glucose, GSH, and ROS) are currently being designed for their application against several diseases, including cancer (Kwon et al., 2015; Liu et al., 2016), with some relevant preclinical outcomes in terms of efficacy (Sun et al., 2017).

Oxidative stress, produced by a disequilibrium between ROS generation and detoxification (Sharma et al., 2007) is a common feature of numerous pathologies, and is promoted by high metabolic demand, oncogenic stimulation, and mitochondrial dysfunction (Pelicano et al., 2004). In inflammatory diseases, the activated leukocytes produce both inflammatory mediators and ROS. Thus, inflammatory diseases are generally characterized by ROS overload (Lu et al., 2017). Similarly in neurodegenerative diseases such as Huntington’s disease, Alzheimer’s disease, and Parkinson’s disease, high oxidative stress is featured (Wang et al., 2015). Therefore, the design of ROS-responsive prodrugs can also improve the selectivity and efficiency of drugs that are applied in these diseases. TK-based ROS-responsive DDS against inflammatory conditions such as inflammatory bowel disease (IBD) (Li et al., 2019) have already been developed and demonstrated to minimize ROS-triggered tissue damage. However, to our knowledge, neither ROS-responsive DDS nor prodrugs against neurological diseases have been designed applying TK-technology so far.

Interestingly, the continuous production of ROS (hydroxyl radical, H_2_O_2,_ and superoxide) by GBM cells is needed for the cells’ growth (Li et al., 2016; Hwang et al., 2018), and the design of DDS that trigger the release of anticancer drugs upon ROS stimulus (known as ROS-responsive DDS) could significantly improve the effectivity of chemotherapeutic agents in GBM as confirmed by a number of studies on ROS-responsive polymeric prodrugs in cancer therapy (Yue et al., 2016; Xu et al., 2017; Pei et al., 2019; Wang et al., 2019b). A plethora of ROS-responsive chemical groups have been developed, for example: polypropylene sulfide, selenium and tellurium, polyoxalate, poly(proline), phenyl boronic ester, and more recently thioketal (TK) were used as linkers for the synthesis of ROS-responsive systems.

Among anticancer drugs, Melphalan (MPH), an alkylating molecule, currently used for the treatment of myeloma, ovarian cancer, breast cancer, neuroblastoma, regionally advanced malignant melanoma, and localized soft tissue carcinoma (Colvin, 2003; Ajazuddin et al., 2013), was inserted into some GBM treatment regimens (Bottom et al., 2000; Gahramanov et al., 2014). Like TMZ, MPH crosses the BBB and is readily taken up by cancer cells making it a good candidate against GBM (Colvin, 2003); however, its poor water solubility (0.1 µg/ml, 25°C) (Ajazuddin et al., 2013) and its non-tumor selectivity represent important drawbacks of its use.

To overcome chemical-physical limitations (i.e., poor solubility) and to increase loco regional and site-specific activity, several strategies are already known and could be exploited. To improve the solubility of chemotherapeutic agents, the conjugation of chemotherapeutic drugs with polyethylene glycol (PEG), known as PEGylation, has a long history with already established strategies present in clinical setting. Beyond the advantage of increasing circulation kinetics by extending residence time in the blood and human safety, (Swierczewska et al., 2015), administrating PEGylated cytotoxic drugs instead of the free drugs could lead to the possibility of by-passing drug efflux, mediated by P-glycoproteins (P-gps). P-gps are one of main reasons for the limited efficacy of chemotherapeutic drugs in GBM, being responsible for efflux events of cytotoxic drugs from the cancer cells (Kirtane et al., 2013) and even at the BBB level. This favorable feature is particularly needed in the case of MPH, with an *in vivo* a circulation half-life of only 75 min (Kuczma et al., 2016).

Furthering this technology by creating a prodrug which is activated as a consequence of a pathological stimulus to improve locoregional and site-specific delivery could be an intelligent approach and has been widely reviewed (Weidle et al., 2014; Taresco et al., 2018; Zeng et al., 2018) and investigated for GBM treatment (Taresco et al., 2018). The innovation would consist of inserting a linker between PEG and a drug that responds to a pathologic stimulus, thus improving the selectivity as well as the effectivity of the drug (Chang et al., 2016). TK linkers are biocompatible linkers which are degraded to thiol-containing groups upon exposure to the most relevant ROS (hydroxyl radical, H_2_O_2_, and superoxide) (Shim and Xia, 2013; El-Mohtadi et al., 2019), and have been recently used in the design of ROS-responsive DDS for the delivery of drugs, siRNA, and DNA in cancer and inflammatory diseases. To our knowledge, TK-based ROS-responsive DDS for the treatment of GBM have not been previously developed (Lee et al., 2013; Zheng et al. 2019). Moreover, few examples of the use of ROS-responsive delivery systems for GBM treatment are reported in the literature, such as, phenyl boronic ester groups (as the ROS-responsive unit) and angiopep-2 peptide (BBB-targeting ligand), for the delivery of siRNA to silence PLK1 and VEGFR2 (Zheng et al., 2019).

Previously, proof-of-concept studies used the biocompatible (mPEG-TK-COOH) (Swierczewska et al., 2015; Regmi et al., 2019) to create an ROS-responsive mPEG-TK conjugate with a fluorescent model drug (Cy5), and demonstrated a stimulus-responsive release of this dye only in brain cancer cells (C6 rat GBM cells) and not in healthy brain cells (rat astrocytes) (Oddone et al., 2019). Based on these results, in this study, we propose to exploit mPEG-TK-COOH polymer, to produce a ROS-responsive antitumor prodrug with MPH, namely mPEG-TK-MPH, for the selective MPH release in GBM cells.

To that end, a physical-chemical characterization as well as *in vitro* efficacy and cytotoxicity studies were performed. Also, because patients are often co-treated with chemical therapeutics and radiation treatment (which could induce ROS production) the potential synergistic effects between the ROS-responsive mPEG-TK-MPH prodrug and X-ray irradiation were explored using rat and human GBM cells in combinatory treatment regimens (Yamamori et al., 2012). These positive results combining the biocompatible mPEG-TK-COOH with MPH to form mPEG-TK-MPH shows great promise in the ability to selectively target GBM cells due to its high ROS levels. This will greatly increase the possibility to further therapeutic options against such a deadly disease. Furthermore, these DDS will readily translate to treatment possibilities for numerous other diseases characterized by high ROS such as inflammatory or neurodegenerative diseases.

## Materials and Methods

### Materials

Methoxy-polyethylene glycol amine (mPEG-NH_2_, Mw 5.000 Da) and mPEG propionic acid (Mw 5.000 Da) were purchased from JenKem Technology and Sigma-Aldrich, respectively. N-hydroxy succinimide (NHS) and N-(3-dimethylaminopropyl) -N-ethyl carbodiimide hydrochloride (EDC. HCl) were obtained from Sigma-Aldrich and used directly. All solvents used, 3-mercaptopropionic acid (3-MPA), dichloromethane (DCM), dimethylformamide (DMF), acetone, hydrogen peroxide (H_2_O_2_), hexane, and methanol were of analytical grade and used without further purification. Melphalan was purchased from Fisher Scientific (Milano, Italy). Ultrapure water, which was used for all the experiments, was provided by a Milli-Q water system (Millipore, Bedford, MA, USA). F12-K and trypsin (TrypLE Select Enzyme [1X]) were purchased from Gibco. Fetal bovine serum (FBS), penicillin-streptomycin, oxidative fluorescent dye, and dihydroethidium (DHE), were purchased from Sigma-Aldrich. DPBS and DMEM High Glucose were purchased from Lonza (Verviers, Belgium).

### Synthesis

#### Synthesis of **TK** Containing Linker (TK-C.L.) and ROS-Responsive mPEG-TK-COOH Polymer

The synthesis of TK-C.L. and mPEG-TK-COOH polymer was performed according to our previous article, without modifications (Oddone et al., 2019). For the synthesis of TK.C.L., a mixture of 3-MPA (49.1 mmol) and anhydrous acetone (98.2 mmol) was stirred for 4 h in HCl (g) atmosphere. The reaction was stopped by placing the mixture on an ice salt bath, and the product was obtained after several washes with hexane and cold water. The product was then characterized by ^1^H NMR and ESI-MS (**Figure S1A**). For the synthesis of mPEG-TK-COOH polymer, the following compounds were dissolved in DCM and stirred for 48 h at ambient temperature: mPEG-NH2 5.000 Da (0.1 mmol), TK-C.L. (1 mmol), EDC. HCl (1.2 mmol), and NHS (1.2 mmol). The product was precipitated with diethyl ether, and after solvent elimination, it was dissolved in a minimal DMF volume and purified by dialysis (MWCO: 3.500 Da) against MilliQ water for 72 h. After dialysis, the product inside the dialysis bag was freeze-dried, and the white powder obtained was characterized by 1H NMR and Bruker Ultraflextreme MALDI-TOF MS/MS (Bruker Daltonics, Bremen, Germany) (**Figure S1B**).

#### Synthesis of ROS-Responsive mPEG-TK-MPH and Non-ROS Responsive mPEG-MPH (Control) Prodrugs

**Scheme 1:**
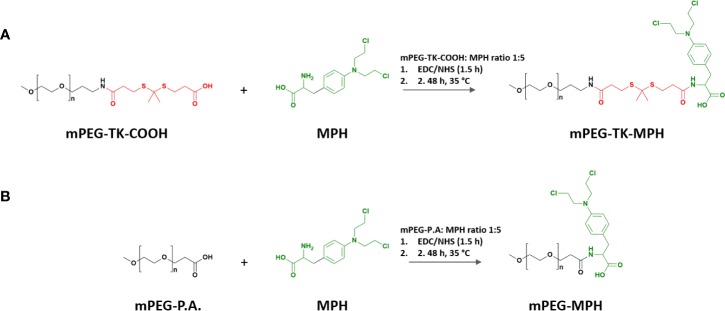
Syntheses of prodrug with MPH. **(A)** mPEG-TK-MPH. **(B)** mPEG-MPH prodrug synthesis.

The terminal carboxylic acid group on mPEG-TK-COOH or mPEG propionic acid (used as control) polymers (6 μmol), were activated with EDC.HCl (60 μmol) and NHS (60 μmol) for 1.5 h in a DMF/DMSO (1:1 v/v) solvent mix. Right after the activation, the reaction was initiated with the addition of MPH (30 μmol) to the mixture and left stirring up to 48 h at 35°C (**Schemes 1A, B**). The reaction was stopped, and the mix dialyzed (MWCO: 3.500 Da) against methanol for 48 h and finally against Milli Q water for an additional 24-h period. At the end of the purification process, the prodrugs were freeze-dried and kept in a desiccator until use. The prodrugs were characterized by ^1^H NMR and MALDI-TOF.

#### Self-Assembly in Water

Either mPEG-TK-MPH or mPEG-MPH prodrugs suspensions in MilliQ water were prepared (0,1–10 mg/ml concentration range). Briefly, an exact amount (10 mg) of either of the prodrugs or their respective precursor polymers (mPEG-TK-COOH and mPEG-P.A., respectively), was suspended in 1 ml of MilliQ water. Then, different prodrug or polymer suspension concentrations were prepared by serial dilutions in MilliQ water for self-assembly ability studies and analyzed by photon correlation spectroscopy (PCS) using Zetasizer Nano ZS (Malvern, UK) and AFM measurements.

### Characterization

#### ^1^H NMR, ESI-MS, and MALDI-TOF

^1^H NMR spectra of TK-C.L. and mPEG-TK-COOH polymer were acquired on Bruker Avance400 NMR (Bruker Biospin, Rheinstetten, Germany) in CDCl_3_. In the case of mPEG-TK-MPH and mPEG-MPH prodrugs, the ^1^H NMR spectra were also acquired on Avance400-Bruker spectrometer in CD_3_OD. For all ^1^H NMR spectra, tetramethylsilane (TMS) was used as an internal standard. The identification of all proton signals in mPEG-TK-MPH and mPEG-MPH was completed after 1D and 2D (COSY) ^1^H NMR analyses.

TK-C.L. mass spectra were acquired with Q-TOF Accurate-Mass G6520A—Agilent Technologies, from which an ESI-MS spectrum in negative mode was obtained. Mass spectra of mPEG-TK-COOH, mPEG-TK-MPH, and mPEG-MPH were acquired with a Bruker Ultraflex TOF/TOF, MALDI-TOF/TOF mass spectrometer.

#### Size and Morphology of Self-Assembled Prodrugs

The mean particle size (Z-average) and the polydispersity index (PDI) of self-assembled prodrug micelles at different prodrug concentrations (10–0.01 mg/ml range) were determined through PCS using a Zetasizer Nano ZS (Malvern, UK; Laser 4 mW He–Ne, 633 nm, laser attenuator automatic, transmission 100–0.0003%, detector avalanche photodiode, Q.E. > 50% at 633 nm) at room temperature. A 10 mg/ml suspension of either of the prodrugs in Milli-Q water was prepared and directly measured. Then, serial dilutions from these prodrug suspensions were prepared and immediately measured after vortex mixing. All measurements were carried out in triplicate.

Atomic force microscopy (AFM) was used to analyze the morphology of self-assembled prodrugs. A selected sample of mPEG-TK-MPH suspended at a concentration of 0.7 mg/ml, was selected to be observed through Atomic Force Microscope (Park Instruments, Sunnyvale, CA, USA), at about 20°C operating in air and in non-contact (NC) mode using a commercial silicon tip-cantilever (high resolution noncontact “GOLDEN” Silicon Canti-levers NSG-11, NT-MDT, tip diameter 5–10 nm; Zelenograd, Moscow, Russia) with stiffness about 40 Nm^−1^ and a resonance frequency around 150 kHz. Briefly, mPEG-TK-MPH dispersed in water at a selected concentration of 0.7 mg/ml, was deposited onto mica surface on a freshly cleaved mica disk (1 cm x 1 cm); 2 min after the deposition, the water excess was removed using blotting paper. The AFM topographical image, representing the amplitude of the vibrations of the cantilever, was obtained with a scan rate of 1 Hz and processed using a ProScan Data Acquisition software.

### MPH Content on mPEG-TK-MPH and mPEG-MPH Prodrugs

MPH content on the prodrugs was expressed as µg of MPH per mg of prodrug and was determined by measuring the absorbance of prodrugs solutions in methanol. The measurement was performed on a spectrophotometer at λ= 300 nm (the maximum absorbance wavelength we obtained with MPH and MPH prodrugs). The MPH content of either of the prodrugs was calculated based on a calibration curve of pure MPH in methanol (linearity in the range between 11.58–74.25 µg/ml; r^2^ = 0.998950).

### *In Vitro* Studies of mPEG-TK-MPH and mPEG-MPH on Astrocytes (Control) Cells and GBM Cells

#### Cell Culture

All cell lines were from ATCC (*American Type Culture Collection*). C6 Rat GBM cells were cultured in F-12 k medium supplemented with 20% FBS and 1% penicillin/streptomycin. Purified newborn rat DI TNC1 primary astrocytes were obtained by the mechanical dissociation method from cultures of cerebral cortex as originally described (McCarthy and de Vellis, 1980). DI TNC1 Rat Astrocyte cells and human GBM cells (U87MG and U251MG cells) were cultured in DMEM High Glucose medium supplemented with 10% FBS and 1% penicillin/streptomycin. All cell lines were maintained in a humidified incubator at 37°C and 5% CO_2_.

#### Determination of the Levels of ROS in Rat GBM and Astrocytes Cells

C6 and DI TNC1 cells were seeded (100,000 cells/ml) on poly-L-lysine (0.1 mg/ml; Sigma-Aldrich) coated glass coverslips in a 24 well plate and incubated at 37°C until 80% confluency was reached. Then, the cells were rinsed with 1X PBS and fixed with 4% paraformaldehyde solution (PFA) in 1X PBS. Cell nuclei were counterstained with DAPI, and coverslips subsequently mounted using Vecta Mount (Vector Laboratories, USA). The cells were observed using a confocal laser-scanning microscope (Zeiss LSM710). CellROX fluorescence of confocal images of C6 and DI TNC1 cells was quantified using ImageJ (National Institutes of Health), by measuring at least 20 cells per condition and cell line.

#### Cytotoxicity Studies of ROS-Responsive and Non-ROS-Responsive Prodrugs on C6 GBM and DI TNC1 Astrocytes Cells

The cytotoxicity of mPEG-TK-MPH and mPEG-MPH prodrugs on C6 cells was evaluated by acquiring cell index vs. time data in real-time, using the xCELLigence RTCA MP instrument (ACEA Biosciences). The experiments were carried out on 16 well E-Plates (ACEA Biosciences), which were coated with poly-l-lysine (PLL). After coating, plates were seeded with C6 or DI TNC1 cells (2 x 10^4^ cells/ml) and put into xCELLigence RTCA MP instrument station, where cell index vs. time curves were recorded. After 24 h of cell seeding, the plates were removed from the instrument, the culture medium was renewed, and cells treated with mPEG-TK-MPH and mPEG-MPH prodrugs at an equivalent concentration of MPH of 11 µM (reported MPH IC50 on C6 cells at 48 h of treatment (Kupczyk-Subotkowska et al., 1997)). Cells were also treated with free MPH and mPEG-TK-COOH (control). Immediately after the addition of the compounds, the plates were put back into XCELLigence station, and the cell index was measured every 5 min up to 48 h. At the end of the experiment, the data were analyzed using the RTCA Data Analysis Software 1.0. The cell index of all treated groups and control groups were normalized to 1 at the time point where the treatment started; normalized cell index vs. time curves were considered for data analysis.

#### Determination of the Levels of ROS in Human GBM Cells

U87 MG and U251 MG cells were grown on glass slides (Ibidi, Martinsried, Germany) by plating at a density of 5 x 10^4^ cells/ml. After 48 h of incubation, the cells were washed with PBS and immediately incubated with DHE fluorescent dye in PBS (5 μM) for 30 min at 37°C. Cells were then washed with PBS and fixed with PFA (4%) for 15 min. The cells were washed with PBS and kept at 4°C until analysis. The cells were observed using a confocal microscope (Zeiss LSM700), and the images obtained were analyzed by Image J. The quantification was performed by measuring the fluorescence of 10 ROI per image in triplicate. Channels used: DAPI (405 nm) and EthD (555 nm).

#### Cytotoxicity Studies of ROS-Responsive and Non-ROS-Responsive Prodrugs on Human GBM Cells

U87 MG and U251 MG cells were seeded on 96 well plates at a density of 5 x 10^4^ cells/ml and kept in an incubator at 37°C for 24 h. The medium was replaced, and cells were treated with mPEG-TK-MPH and mPEG-MPH prodrugs, at equivalent concentrations of MPH (concentration range: 10–1,000 µM) as well as free MPH. After 48 h of treatment, the medium was removed, the cells were washed with DPBS and then incubated for 3 h at 37°C in culture medium containing Resazurin (44 μM). Finally, the fluorescence was measured on a plate reader (CLARIOstar) at λexc= 545 nm/λem= 600 nm. Relative cell viability was expressed as a percent where 100% was set based on the non-treated control cells.

#### Influence of X-Ray Irradiation on ROS-Responsive and Non-ROS-Responsive Prodrugs Cytotoxicity on Human GBM Cells

U87 MG and U251 MG cells were seeded in 96 well plates at a density of 5 x 10^4^ cells/ml. After 24 h of incubation, the cells were irradiated with X-ray (Edimex Faxitron) at the dose of 4 Gy. Immediately after being irradiated, cells were treated with free MPH (1,000 µM), mPEG-TK-MPH, and mPEG-MPH prodrugs at an equivalent concentration of 1,000 µM. After 24 h, a second irradiation round (4 Gy dose) was applied to the cell plates. Finally, at the end of the experiment (48 h counted from the first irradiation dose), the cell viability of cells was indirectly measured again by the resazurin method (described in section 2.5.5).

### Statistics

All data are shown as the mean of at least three experiments ± SD. GraphPad Prism 5 was used for statistical analyses. For pairwise comparisons, unpaired t-test, one-way, and two-way ANOVA with Bonferroni post-hoc test analysis were performed.

## Results and Discussion

### mPEG-TK-MPH and mPEG-MPH Synthesis and Characterization

Both prodrugs (mPEG-TK-MPH and control mPEG-MPH) (**Schemes 1A, B**) were obtained as a pale-yellow powder, with product yields over 90% (94 and 93%, respectively). The absence of free MPH, and thus the purity of both prodrugs was confirmed by RP-HPLC (data not shown). The MPH content of mPEG-TK-MPH and mPEG-MPH prodrugs was 56.3 ± 1.1 μg/mg and 63.6 ± 4.9 μg/mg, respectively.

**Figure 1:**
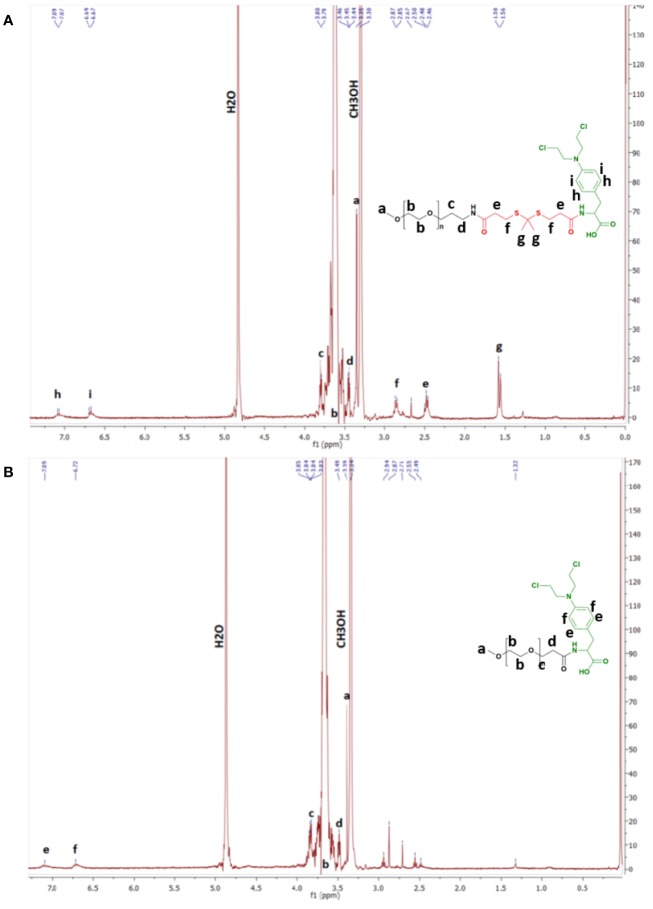
^1^H NMR spectra in CD_3_CD. **(A)** mPEG-TK-MPH and **(B)** mPEG-MPH.

^1^H NMR spectra of either of the prodrugs show the presence of chemical shifts ascribed to protons pertaining to the benzenic group of MPH (6.69 and 7.09 ppm) (**Figures 1A, B** and **Figure S2**). In addition, the chemical shift ascribed to the methyl group protons of TK (1.58 ppm) in the mPEG-TK-MPH spectrum can be observed. The mean molecular weight of mPEG-TK-MPH and mPEG-MPH prodrugs, obtained by MALDI TOF, were 5374.8 and 5300.7 g/mol, respectively, right-shifted in the spectra with respect to the MW of their starting polymers: mPEG-TK-COOH (5233.1 g/mol) and mPEG-P.A. (5168.5 g/mol). The characterization results confirmed the covalent conjugation of MPH to both mPEG polymers (mPEG-TK-COOH and mPEG-PA).

### Size and Morphology of Self-Assembled mPEG-TK-MPH and mPEG-MPH Prodrug Micelles

**Figure 2:**
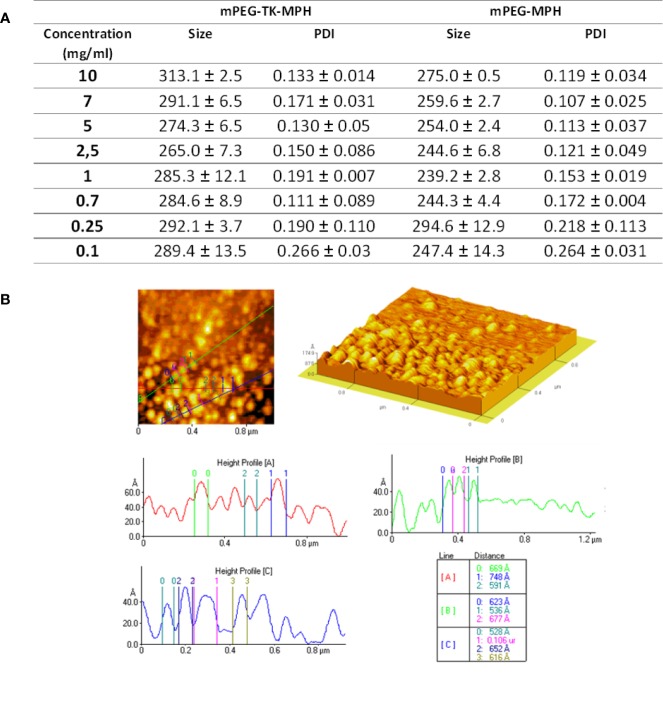
**(A)** Mean size and PDI of prodrug micelles at different prodrug concentrations. **(B)** Representative AFM image and height profile of mPEG-TK-MPH dispersed in water at a concentration of 0.7 mg/ml.

Since mPEG-TK-MPH and mPEG-MPH prodrugs have a hydrophilic portion (mPEG) covalently linked to a hydrophobic molecule (MPH), they might self-assemble in aqueous solution. This possibility was investigated by PCS and AFM. Therefore, a study of size variation and poly-dispersity as a function of the prodrug aqueous concentration was performed in milliQ water a range of 0.1–10 mg/ml (**Figure 2A**). At any concentration, from 10 to 0.1 mg/ml, the mean size of mPEG-TK-MPH self-assembly micelles ranged from 260 to 300 nm.

On the contrary, PDI values, indicating the homogeneity of the samples, varied; under 0.25 mg/ml, both prodrugs formed assemblies with extremely high PDI values demonstrating a non-homogenous particle distribution, while from 0.25 to 10 mg/ml, all PDI values were acceptably low. The lowest PDI values (0.111), indicating the highest sample homogeneity, was recorded at 0.7 mg/ml.

mPEG-TK-COOH did not show monodisperse compositions with high PDI values at all concentrations (**Figure S3**); this was an expected result considering that due to its non-amphipathic nature this polymer does not self-assembly. Since mPEG-TK-MPH suspended at the concentration of 0.7 mg/ml showed the lowest PDI index, this concentration was selected to further characterize the morphology of self-assembled prodrugs by AFM. The analyzed sample of mPEG-TK-MPH suspended at 0.7 mg/ml demonstrated spherical structures sizing around 100 nm (around 50–80 Ångström) that can be ascribed to prodrug micelles (**Figure 2B**).

The discrepancy in size among mean hydrodynamic size and the mean size obtained by AFM could be related to the fact that the effective hydrodynamic size includes the solvent surface layers of nanocarriers, which are no longer present in dried samples used for high-resolution microscopic techniques (Oddone et al., 2016). This result is in accordance with other research works in which particle size measured using PCS differed from that measured by TEM (Souza et al., 2016; Wang et al., 2019b).

### Cytotoxicity Studies of mPEG-TK-MPH and mPEG-MPH on Murine GBM Cells (C6) and Healthy Astrocyte Cells (DI TNC1)

The selectivity of ROS-responsive mPEG-TK-MPH prodrug for GBM versus healthy cells *in vitro* was evaluated on rat GBM cells (C6 cells) and “healthy” primary rat astrocytes cells (DI TNC1). The levels of ROS produced intrinsically by these cells were comparatively measured (data not shown), highlighting that C6 cells produced significantly higher levels of ROS than DI TNC1 cells, in agreement with previous studies (Oddone et al., 2019).

**Figure 3:**
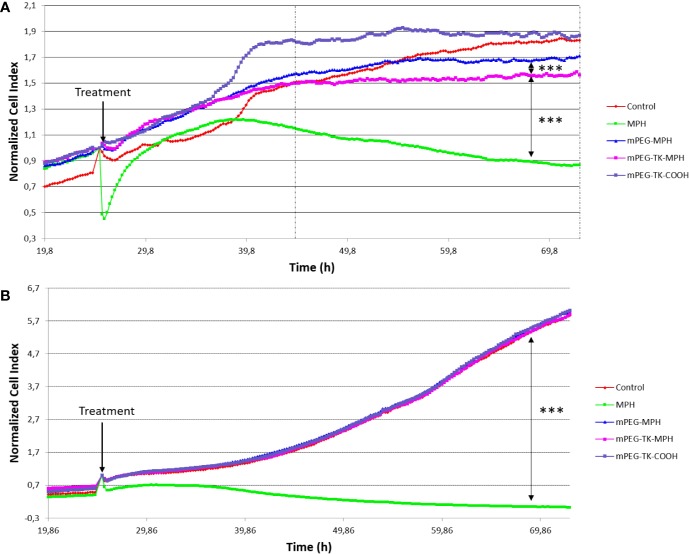
Normalized Cell Index curves of C6 (rat GBM cells) and DI TNC1 (Astrocyte cells). Both types of cells were treated with mPEG-TK-COOH (polymer control), free MPH (11 µM), mPEG-TK-MPH, and mPEG-MPH prodrugs at an equivalent MPH concentration (11 µM) for 48 h. **(A)** C6 cells. Statistics: One-way ANOVA tests with Bonferroni post-test. Between the dashed lines is indicated the time period in which the differences between treatments are significant and are depicted in the graph (***p ≤ 0.001). Time range: 19.5 h from the addition of the treatments, up to the end of the experiment (48 h). **(B)** DI TNC1. The significant difference depicted in the graph was calculated considering the full time range from the addition of the different treatments to the end of the experiment (48 h).

Cytotoxicity of MPH-based prodrugs on C6 and D1 TNC1 cells were evaluated showing that when C6 GBM cells (**Figure 3A**) were treated with either mPEG-TK-MPH prodrug or free MPH, a significant cell growth inhibition in comparison to the control group was observed (**Figure 3A**). Furthermore, the cell growth of C6 cells treated with mPEG-TK-MPH was significantly inhibited in comparison to that of cells treated with mPEG-MPH. To answer a question about a possible mechanism which could justify high cytoxicity of mPEG-TK-MPH with respect to mPEG-MPH, we stressed that when the level of ROS in endosomes is elevated, a ROS response can induce drug release and endosomal escape (Sun et al., 2017). As we previously confirmed that Cy5 from mPEG-TK-Cy5 conjugate (which was obtained starting from the same ROS-responsive polymer, mPEG-TK-COOH) is endocytosed by C6 GBM cells, and then released inside these cells (Oddone et al., 2019). We therefore imagine that after the endocytosis of self-assembled mPEG-TK-MPH micelles, the presence of ROS inside the endosomes can trigger the cleavage of TK bonds from the prodrugs with subsequent MPH release. Afterward, the released MPH can escape from the endosomes and then diffuse into the cell nucleus, where MPH finally will inhibit cell proliferation by inducing DNA inter-strand cross-links (ICL) (Kühne et al., 2009; Xiong et al., 2015).

Nevertheless, the cytotoxicity of the free drug was significantly higher in comparison to either of the prodrugs in cancer cells (**Figure 3A**), but also in healthy cells (**Figure 3B**). This may be due to a higher cell uptake favored by L-type amino acid transporters (LATs) (Kühne et al., 2009) (as L-type amino acid transporter 1, LAT1), which transport neutral amino acids, including leucine. This transporter is present also in glioma cells, including C6 (Lin et al., 2004; Nawashiro et al., 2005; Nawashiro et al., 2006; Kuczma et al., 2016), and can promote MPH active transport into cells (Lin et al., 2004). In addition, taking into consideration its hydrophobic nature, passive transport of the free form of the drug cannot be excluded (Kühne et al., 2009).

In contraposition, a decreased activity of MPH when in mPEG-TK-MPH prodrug was experienced, we hypothesized that these prodrugs are internalized by endocytosis, probably by fluid-phase endocytosis, as previously observed with mPEG-Cy5 conjugates (Oddone et al., 2019). As these prodrugs were not modified with any targeting ligand for GBM cells, their cell uptake might not be as high as it could be by exploiting receptor-mediated endocytosis (Zhang et al., 2019). Apart from this aspect, since TK cleavage was previously confirmed to be dependent not only on ROS amount but also of the exposed time to ROS (Oddone et al., 2019), it can be argued that the time needed to release MPH from the prodrug and/or micelle-like architecture may impact release kinetics and, therefore increase or decrease the rate of GBM cell death induction.

Regarding “healthy” DI TNC1 astrocyte cells, the prodrugs did not influence cell growth (**Figure 3B**), indicating the inability of triggering MPH drug release from mPEG-TK-MPH in cells with physiological levels of ROS, which are lower than in other glioma (C6) cancer cells (Oddone et al., 2019). On the other hand, control treatment with free MPH led to a pronounced inhibition of cell growth in both cell lines. This could be ascribed to the expression of LAT1 transporters in all cells (Sampaio-Maia et al., 2001).

At the end of the experiment, concerning prodrugs administrations, the normalized cell index of free MPH treated cells was ~245-fold lower in healthy cells, describing a very low selectivity toward cancer versus healthy cells as described by several studies, and the frequent side-effects of free MPH (Kühne et al., 2009; Kuczma et al., 2016). Remarkably, from analysis of the normalized cell index ratio between mPEG-TK-MPH and free MPH in both types of cells (**Figures 3A, B**), we can conclude that mPEG-TK-MPH was significantly favorable in terms of safety treated in healthy cells with minimal toxicity and showed good selectivity in the case of cancer cells, with normalized cell index ratios at 48 h treatment of (295 vs 1.8, respectively).

### *In Vitro* Studies of mPEG-TK-MPH and mPEG-MPH Prodrugs on Human GBM Cells

Apart from performing cytotoxicity studies with the prodrugs on rat GBM cells, studies were also performed using two human GBM cell lines: U87 MG and U251 MG, derived from grade IV GBM tumors. These cell lines are frequently used for *in vitro* drug screening previous to *in vivo* tests in animal models (Lenting et al., 2017).

**Figure 4:**
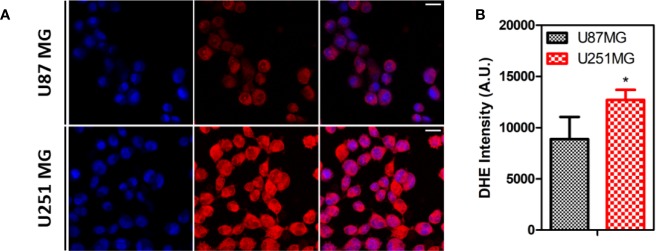
ROS levels on human GBM cells. **(A)** Representative images of U87MG and U251MG cells treated with DHE. **(B)** Quantification of DHE intensity on human GBM cells Statistics: Unpaired t-test (*p < 0.05).

The intrinsic levels of ROS produced by these cells were indirectly measured by incubating the cells with DHE, a dye that can detect cytosolic superoxide, peroxynitrite, and hydroxyl radical (Wojtala et al., 2014). The results indicate a higher level of ROS in U251 MG in comparison to U87 MG cells (**Figure 4**).

**Figure 5:**
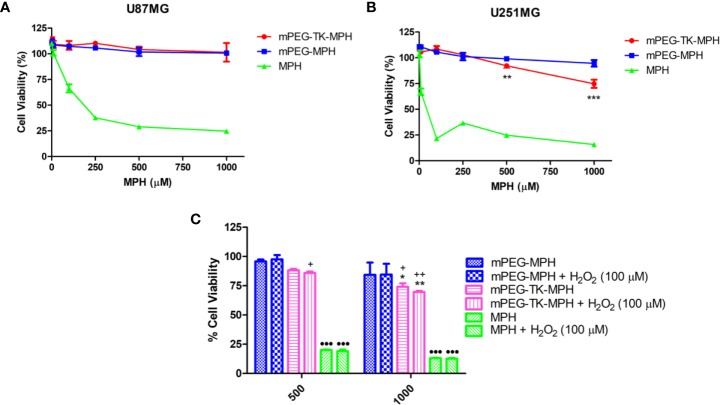
Cytotoxicity studies on human GBM cells. **(A)** U87 MG cells treated with increasing concentration of either free MPH, mPEG-TK-MPH, or mPEG-MPH prodrugs at equivalent MPH concentrations for 48 h. **(B)** U251 MG cells treated with increasing concentration of either free MPH, mPEG-TK-MPH, or mPEG-MPH prodrugs at equivalent MPH concentrations, for 48 h. Statistics: Two-way ANOVA tests with Bonferroni post-test. Comparisons: mPEG-MPH vs. mPEG-TK-MPH (**p ≤ 0.01 and ***p ≤ 0.001). **(C)** Co-incubation of U251 MG cells treated with free MPH, mPEG-TK-MPH, or mPEG-MPH prodrugs, with H_2_O_2_ (100 µM). Statistics: Two-way ANOVA tests with Bonferroni post-Comparisons: mPEG-MPH vs. mPEG-TK-MPH and mPEG-TK-MPH + H_2_O_2_ (*p ≤ 0.05 and **p ≤ 0.01), mPEG-MPH + H_2_O_2_ vs. mPEG-TK-MPH and mPEG-TK-MPH + H_2_O_2_ (^+^p ≤ 0.05 and ^++^p ≤ 0.01), mPEG-MPH or mPEG-TK-MPH (w/and w/o H_2_0_2_) vs. MPH and MPH + H_2_O_2_ (^•••^p ≤ 0.001).

Next, the cytotoxicity of mPEG-TK-MPH and mPEG-MPH prodrugs on these human GBM cells was assayed by treating the cells with different concentrations of free MPH, or either of the prodrugs (at an equivalent concentration of MPH), or an equivalent volume of cell media (which was used as control values and set as 100% for comparison) for 48 h. Regarding U87 MG cells, neither the ROS-responsive mPEG-TK-MPH nor the non-ROS responsive mPEG-MPH prodrugs showed to be cytotoxic at the MPH concentration range and time used in this experiment (**Figure 5A**). On the contrary, at 500 and 1,000 µM equivalent concentrations of MPH, only mPEG-TK-MPH showed to be cytotoxic on U251 MG cells (**Figure 5B**) with a significant reduction on cell viability in comparison to the control prodrug mPEG-MPH.

As it was observed for rat GBM cells, the cytotoxicity obtained with free MPH was significantly higher than the observed with either of the prodrugs (**Figures 5A, B**), with IC50s of 89 and 16 µM in U87 MG and U251 MG cells, respectively. This result could be related to the higher sensitivity of these kind of cells to MPH due to the overexpression of LAT1 transporter that is responsible for the active transport of MPH into proliferating tumor cells including U87 MG and U251 MG.

As higher levels of ROS were found in U251 MG in comparison to U87 MG, we hypothesize that the higher cytotoxicity of mPEG-TK-MPH in U251 MG might be related to the higher intrinsic intracellular levels of ROS produced by these cells.

An alternative explanation for the lower cytotoxicity of mPEG-TK-MPH in comparison with free MPH might lie on a possible insufficient TK cleavage by ROS. Therefore, to investigate if, by increasing the level of ROS, the cytotoxicity of ROS-responsive mPEG-TK-MPH can be increased, we forced experimental conditions by adding H_2_O_2_ as a ROS enhancer. We, First, different H_2_O_2_ concentration on either of the GBM cells were tested in order to choose a non-toxic concentration (**Figure S4**). A non-toxic H_2_O_2_ concentration of 100 µM was used to measure the intracellular levels of ROS in U87 MG and U251 MG cells after different H_2_O_2_ incubation times (**Figure S5**). We observed that the levels of ROS in U87MG cells treated with H_2_O_2_ at different times did not increase (**Figure S5A**). In contrast, the levels of ROS in U251 MG cells were particularly increased after 24 h incubation with H_2_O_2_, suggesting that H_2_O_2_ can be used to force the release of higher amounts of MPH in these cells. Therefore, next, we tested the effect of H_2_O_2_ on the cytotoxicity of mPEG-TK-MPH in U251 MG cells. Free MPH, mPEG-TK-MPH or mPEG-MPH, were co-incubated with 100 µM H_2_O_2_ for 48 h. Remarkably, at the equivalent MPH concentrations tested (500 and 1,000 µM), we observed (**Figure 5C**) a more evident cytotoxic effect of mPEG-TK-MPH corresponding to a slight reduction of cell viability in H_2_O_2_ co-incubated cells, compared to standard cell conditions. This result, therefore, confirmed that when the intracellular concentration of ROS increases, the release of MPH from mPEG-TK-MPH and its cytotoxic activity improve.

### Evaluation of the Potential Synergistic Effect of X-Ray Irradiation on Human GBM Cells Treated With mPEG-TK-MPH and mPEG-MPH Prodrugs

In U251 human GBM cells, mPEG-TK-MPH showed to be cytotoxic at concentrations higher than 500 µM. Since an improved cytotoxicity of mPEG-TK-MPH prodrug after additional enhancement of ROS using H_2_O_2_ was noticed, it was hypothesized that the physiological levels of ROS in GBM cells might not be enough to induce TK linker cleavage from the ROS-responsive prodrug (mPEG-TK-MPH). Radiotherapy, defined as the use of ionizing radiation (IR) in therapy, was reported to be able to stimulate and increase mitochondrial-related ROS (Kawamura et al., 2018). Given that the current clinical setting used for GBM treatment is based on concomitant chemotherapy and radiotherapy to improve patient survival (McKelvey et al., 2018), the effect of combining ROS-responsive prodrug treatment with X-ray, a type of IR, on human GBM cells was explored.

Notably, only a few studies report ROS-responsive DDS used in combination with a different type of IR (γ-ray). For instance, ROS-responsive selenium-based micelles containing doxorubicin demonstrated to have a synergistic antitumor effect on hepatocellular carcinoma cells at 5 Gy radiation dose (Ma et al., 2011). This result could be explained due to a higher sensitivity of hepatocellular carcinoma cells in promoting ROS induction after irradiation. Similarly, tellurium-based micelles (Cao et al., 2015) demonstrated to respond to an even lower radiation dose (2 Gy).

**Figure 6:**
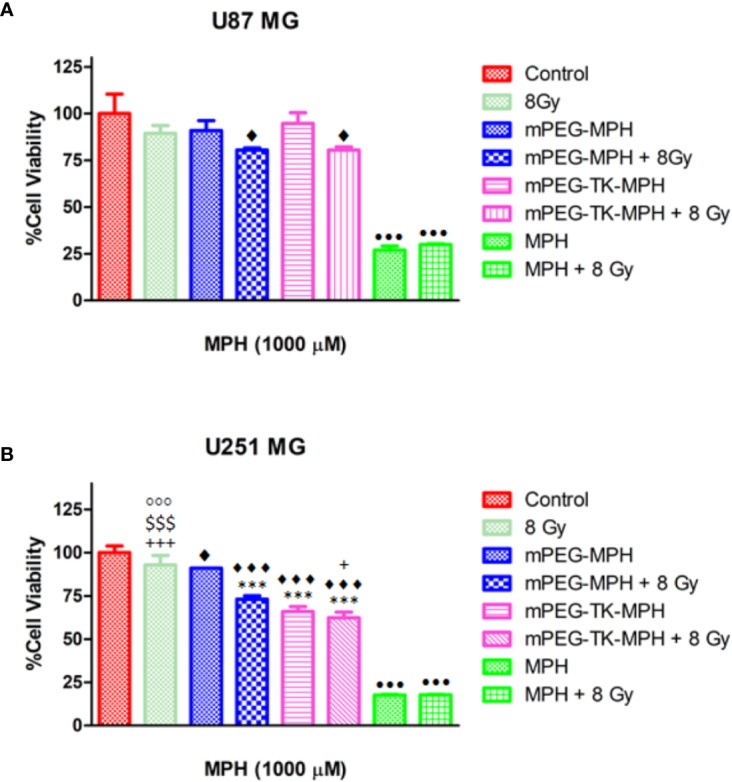
Influence of X-ray irradiation on human GBM cells treated with mPEG-TK-MPH and mPEG-MPH. **(A)** U251 MG cells and **(B)** U87 MG cells. Statistics: One-way ANOVA tests with Bonferroni post-test. Comparisons: Control vs. mPEG-MPH, mPEG-MPH + 8 Gy, mPEG-TK-MPH and mPEG-TK-MPH + 8Gy (^♦^p ≤ 0.05 and ^♦♦♦^p ≤ 0.001); mPEG-MPH vs. mPEG-MPH + 8 Gy, mPEG-TK-MPH and mPEG-TK-MPH + 8 Gy (***p ≤ 0.001); mPEG-MPH + 8 Gy vs. 8 Gy, mPEG-TK-MPH and mPEG-TK-MPH + 8Gy (^+^p ≤ 0.05 and ^+++^p ≤ 0.001), mPEG-TK-MPH vs. 8 Gy and mPEG-TK-MPH + 8 Gy (^$$$^p ≤ 0.001); 8Gy vs. mPEG-TK-MPH + 8Gy (^◦◦◦^p ≤ 0.001); MPH and MPH + 8 Gy vs. Control and all other treatments (^•••^p ≤ 0.001).

As a consequence, a protocol in which GBM cells received an overall X-ray dose of 8 Gy at 2 times (4 Gy as an initial dose and 4 Gy after 24 h) was performed, which showed that irradiated U87 MG and U251 MG cells did not suffer from any cytotoxicity (**Figures 6A, B**). Then, the cytotoxicity of mPEG-TK-MPH and mPEG-MPH at 1,000 µM equivalent concentration of MPH (MPH concentration in which we observed the highest cytotoxicity of mPEG-TK-MPH on U251 MG cells) on irradiated human GBM cells was tested. Remarkably, viability tests showed an increase in cytotoxicity upon prodrug (mPEG-MPH and mPEG-TK-MPH) treatment and irradiation (**Figures 6A, B**) in comparison to control cells on both U87 MG and U251 MG cells.

As shown in previous experiments by Gill and Vallis, the cytotoxic effect obtained by the combination of a chemotherapeutic drug and IR is higher than predicted due to the additive effect of the therapies, meaning that the chemotherapeutic drug can be classified as a radiosensitizer. Since many potent radiosensitizers are DNA-damaging agents (Gill and Vallis, 2019), MPH could be reasonably investigated as radiosensitizer agent by increasing IR cytotoxicity. While for free MPH there was no difference observed among irradiated and non-irradiated cells (**Figures 6A, B**), the high cytotoxicity of free MPH on any of the cell lines might explained why at the dose applied in these experiments a synergistic effect was not evident; however, the higher cytotoxicity seen with mPEG-MPH and mPEG-TK-MPH on U87 MG and with mPEG-MPH on U251 MG irradiated cells, might be due to a synergistic effect with MPH acting as a radiosensitizer. The lack of toxicity in U87MG cells is likely that the ROS status, even in the presence of beam radiations, does not reach the required amount to cleave TK group on mPEG-TK-MPH prodrug.

In the case of U251 MG cells, the cytotoxicity of mPEG-TK-MPH treated, and irradiated cells was significantly higher in comparison to mPEG-MPH treated and irradiated cells (**Figure 6B**). Nevertheless, the slight reduction on cell viability of mPEG-TK-MPH treated and irradiated cells in comparison to only mPEG-TK-MPH (without irradiation) was not significant. This result might indicate that at the X-ray dose received, the production of ROS was not enough strong to induce TK bond cleavage and MPH release from mPEG-TK-MPH to achieve higher cytotoxicity.

Thus, we can conclude that the radiation conditions used were not capable to produce sufficient concentrations of ROS that trigger the release of active MPH. Possible instant or cumulative effect on ROS production could be obtained by using higher X-ray dose or X-ray dose regimen (e.g. fractionation, kinetic) respectively. In addition, it could be interesting to investigate in further experiments alternative IR therapy such as alpha and beta radiation, that might have a different impact on ROS production and therefore, on TK cleavage.

Since the local administration of drugs has been investigated as it offers the possibility to bypass the BBB and blood brain tumor barrier (BBTB), concentrating higher amounts of drug in malignant tumors (Nam et al., 2018), it would be interesting to provide a locally sensitive strategy which would make it possible to have synergism with other actions including radiotherapy for the cleavage and drug activity. For the moment, pilot clinical studies using intravenous MPH have not shown any significant impact (Anderson et al., 2015). Thus, this new activatable ROS-responsive prodrug agent is interesting for local administration, or alternatively could be further functionalized with a BBB-targeting ligand, such as our previous work with the g7-peptide, promoting its passage into the brain for intravenous applications (Cox et al., 2019) and should be further explored.

## Conclusions

With this study, we demonstrated that ROS-sensitive mPEG-TK-MPH displayed a higher anticancer activity and cytotoxicity than the non-ROS sensitive prodrug mPEG-MPH in rat C6 and human U251 MG GBM cells. Furthermore, we demonstrated a higher safety profile of ROS-sensitive mPEG-TK-MPH since it did not induce any cytotoxicity in healthy cells (DI TNC1 astrocyte cells). Therefore, due to their ability to specifically deliver drugs upon ROS stimulus with increased effectivity and selectivity for cancer over healthy cells, the application of TK-technology in the design of prodrugs could be considered as a promising approach for the development of future therapeutics against GBM. In addition, we investigated the potential synergistic activity of X-ray radiation on human GBM cells, observing that both prodrugs were cytotoxic U87MG cells only when also irradiated. On U251MG cells, both prodrugs demonstrated to be cytotoxic, but in the case of mPEG-TK-MPH, the cytotoxicity was comparable to non-irradiated mPEG-TK-MPH treated cells. Thus, the observed cytotoxicity effect was probably due to the irradiation increasing MPH toxicity, but not to an additional cleavage of TK groups by X-ray generated ROS. Since in this work we could demonstrate that mPEG-TK-MPH respond to intracellular ROS in GBM cells, we are planning to investigate *in situ* the stimuli or local combinations capable of causing the expected MPH release and antitumor effect.

Furthermore, since the ROS-response technology of mPEG-TK-MPH was demonstrated to work in GBM cells expressing high ROS levels, large improvements in terms of cytotoxicity could be realistically obtained in clinical translation. This study could be a starting point for a rationale design of innovative and tailored ROS-responsive prodrugs, capable of not only improving drug solubility and mediating selective ROS-triggered drug release but also to improve the transport of highly cytotoxic drugs with poor cell permeation. In addition, other diseases such as those with a pro-inflammatory component, and neurodegenerative diseases are featured by high levels of ROS as well and the use of TK-technology might be translated to these diseases by coupling a selected drug to the mPEG polymer though a TK linkage.

## Data Availability Statement

All datasets generated for this study are included in the article/**Supplementary Material**.

## Author Contributions

NO has participated in all procedures of the article. FB, EG, AG, JD, GT, and BR have contributed in supervision activities, and writing and editing of the article. MM, FD, AJ, FF, MV, and JD have participated in investigation and funding acquisition.

## Funding

NO was supported by PhD School in Clinical and Experimental Medicine, University of Modena and Reggio Emilia. The authors thank Sarah Hudson and Dr. Edel Durack from Biopoint, Bernal Institute, University of Limerick for the MALDI-TOF/TOF mass spectrometry measurements. AG acknowledges networking support by the University of Limerick Cancer Network (ULCaN).

This work was additionally supported by the French national research agency (ANR) through the LabEx IRON < < Innovative Radiopharmaceuticals in Oncology and Neurology>> as part of the French government “Investissements d’Avenir” program (ANR-11-LABX-0018) and related to: i) the ANR and Italian Ministry of Health under the frame of EuroNanoMed III (project GLIOSILK), ii) to the PL-BIO 2014-2020 INCa (Institut National du Cancer) consortium MARENGO < < MicroRNA agonist and antagonist Nanomedicines for Glioblastoma treatment: from molecular programmation to preclinical validation>>, and iii) to the MuMoFRaT project < < Multi-scale Modeling & simulation of the response to hypo-Fractionated Radiotherapy or repeated molecular radiation Therapies>> supported by “La Région Pays-de-la-Loire” and by the Cancéropôle Grand-Ouest (Vectorization, imaging and radiotherapies network). This work was also supported by the NanoFar program <<European doctorate in nanomedicine and pharmaceutical innovation>> (Erasmus Mundus Joint Doctorate) funded by EACEA and by the NanoFar+ program <<International strategy>> funded by “La region des pays de la Loire”.

Moreover, the work was supported by MAECI GRANT PGR00819 “Nanomedicine for BBB-crossing in CNS oncologic pathologies” and COST Action CA 17140 “Cancer Nanomedicine from the Bench to the Bedside” supported by COST (European Cooperation in Science and Technology). This work was also partially supported by Por Fesr 2014-2020 Regione Emilia-Romagna, Asse 1, Azione 1.2.2, Mat2Rep Project.

## Conflict of Interest

The authors declare that the research was conducted in the absence of any commercial or financial relationships that could be construed as a potential conflict of interest.
