# Supplementary material for: Synthesis, Characterization, and In Vitro Studies of an Reactive Oxygen Species (ROS)-Responsive Methoxy Polyethylene Glycol-Thioketal-Melphalan Prodrug for Glioblastoma Treatment
Source: Front Pharmacol. 2020 May 4;11:574. doi: 10.3389/fphar.2020.00574 (PMC7212708; doi:10.3389/fphar.2020.00574)
Supplement: Supplementary file 1 [file DataSheet_1.docx]

Supplementary Material

**A.**


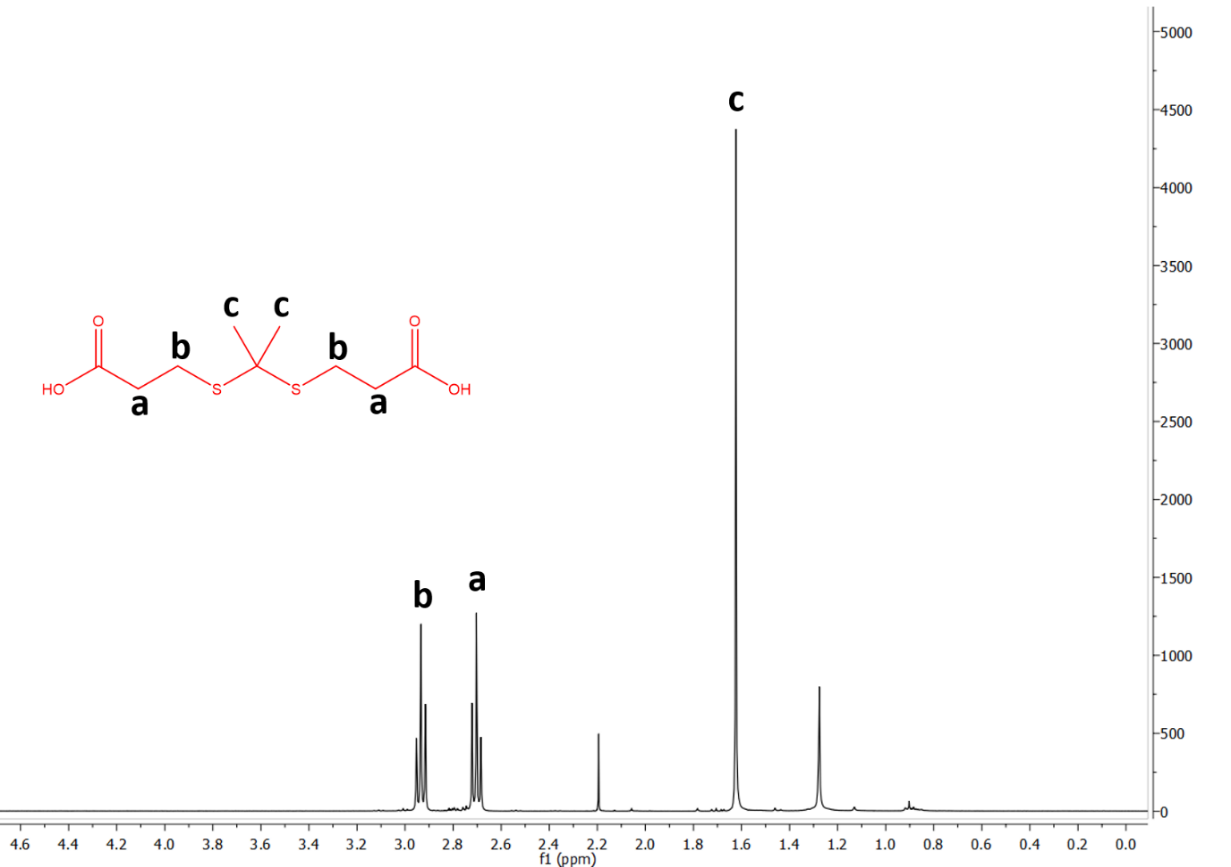


**B.**


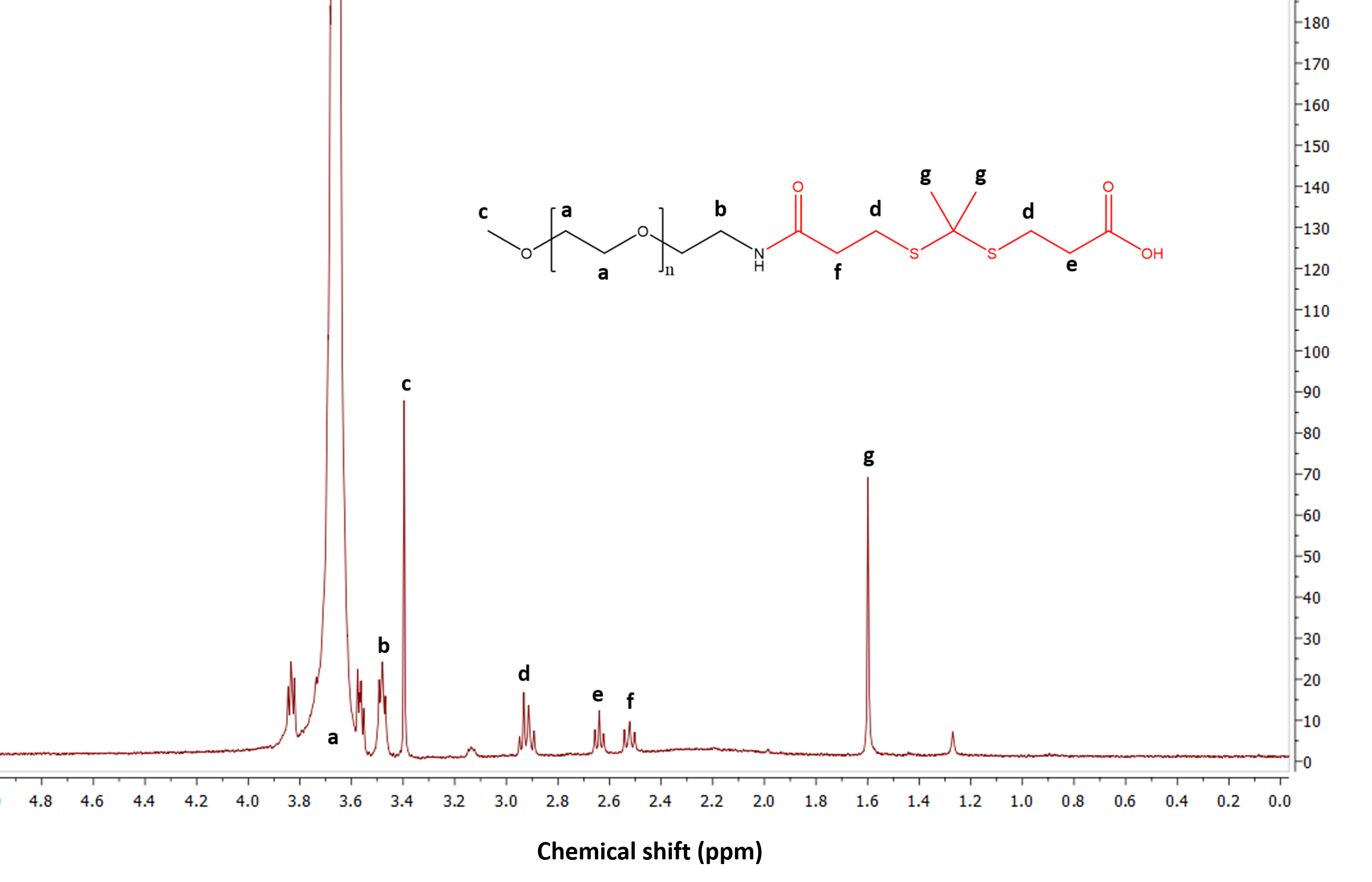


**Figure S1.** A. 1H NMR spectra of TK-C.L. and B. mPEG-TK-COOH polymer.


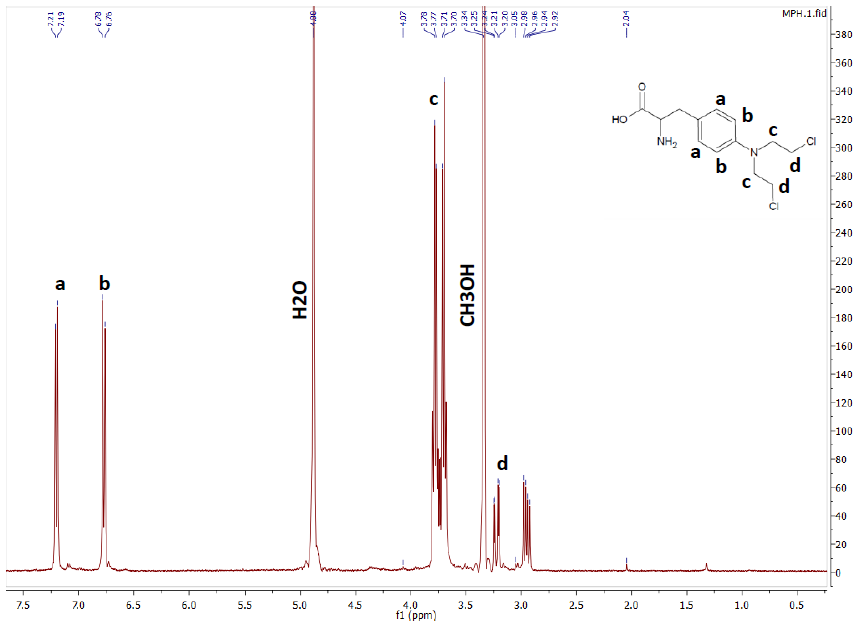


**Figure S2.** ^1^H NMR MPH in CD_3_CD

**Figure S3. Mean size and PDI of mPEG-TK-COOH in water at 10-0.1 mg/mL range.**


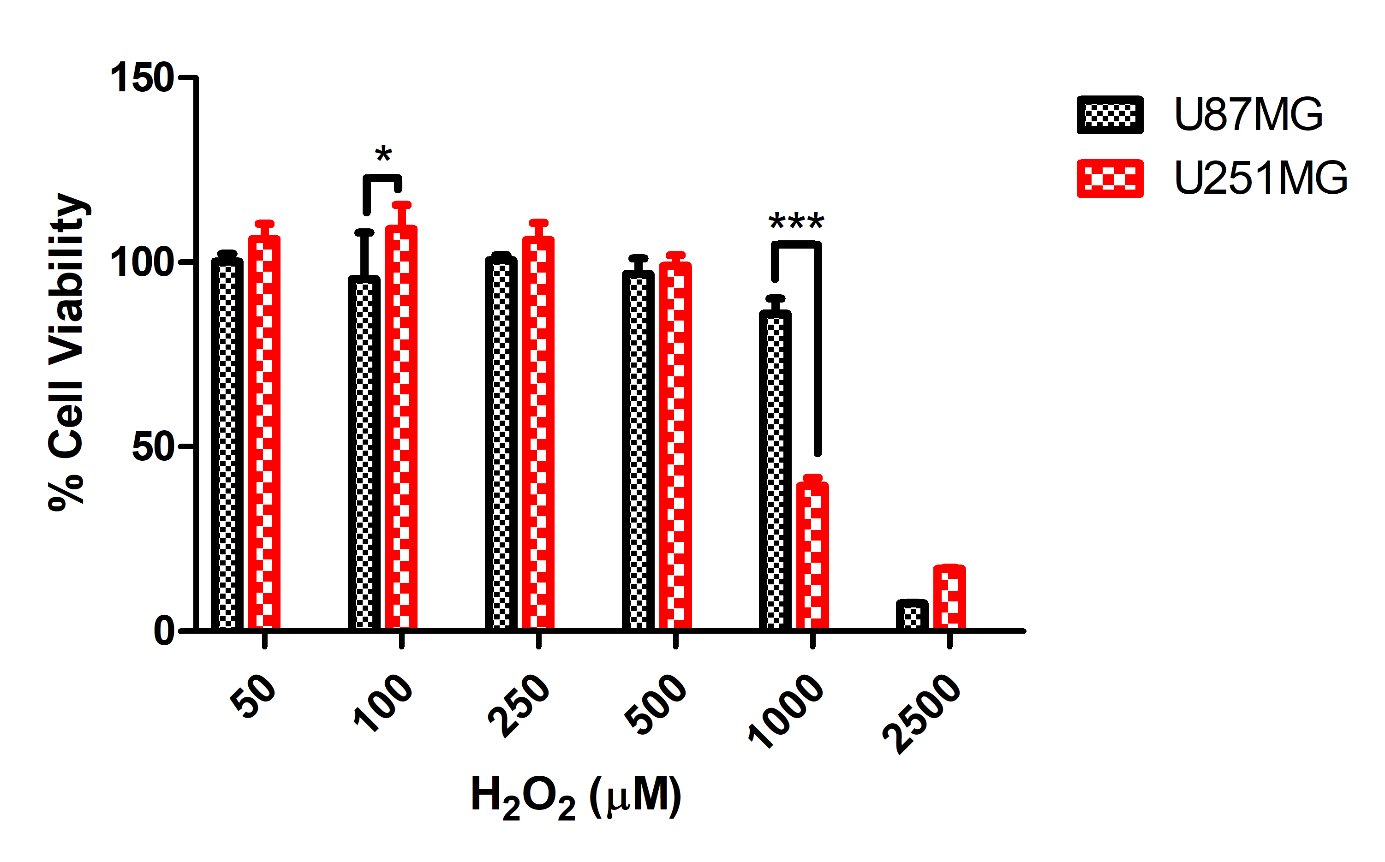


**Figure S4:** Cytotoxicity study performed by incubating U87 MG and U251 MG cells with increasing H_2_O_2_ concentrations. 2-way ANOVA, Bonferroni post test (*p≤0.05, **p≤0.05). The H_2_O_2_ calculated IC 50 for U87MG and U251 MG cells were of 1252 and 783.6 µM, respectively.


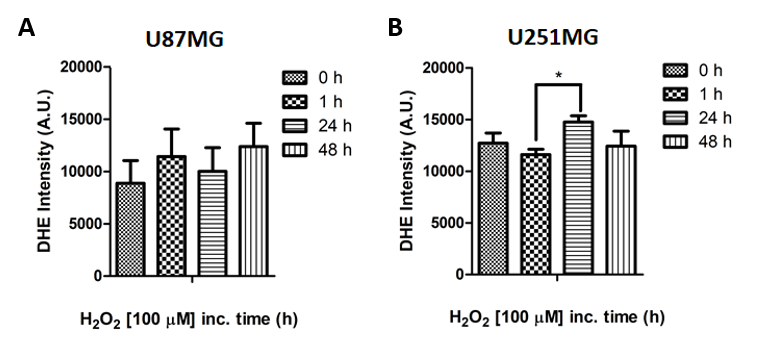


**Figure S5:** DHE intensity of human GBM exposed to H_2_O_2_ for different incubation times. **A.** U87MG and **B.** U251MG cells. 1-way ANOVA, Bonferroni post test (*p≤0.05).
